# Eosinophils in Colorectal Cancer: Emerging Insights into Anti-Tumoral Mechanisms and Clinical Implications

**DOI:** 10.3390/ijms25116098

**Published:** 2024-06-01

**Authors:** David Lopez-Perez, Belen Prados-Lopez, Julio Galvez, Josefa Leon, Angel Carazo

**Affiliations:** 1 Department of Pharmacology, Faculty of Pharmacy, University of Granada, 18012 Granada, Spain; 2 Research Unit, Instituto de Investigación Biosanitaria de Granada (ibs.GRANADA), 18016 Granada, Spain; 3 Centro de Investigación Biomédica en Red para Enfermedades Hepáticas y Digestivas (CIBER-EHD), Center for Biomedical Research, University of Granada, 18012 Granada, Spain; 4 Unidad de Gestión Clínica de Aparato Digestivo, Hospital Universitario San Cecilio de Granada, 18016 Granada, Spain; 5 Unidad de Gestión de Microbiología, Hospital Universitario San Cecilio de Granada, 18016 Granada, Spain

**Keywords:** eosinophils, colorectal cancer, tumor microenvironment, tumor evasion, mucosal immune responses

## Abstract

Eosinophils are myeloid effector cells whose main homing is the gastrointestinal tract. There, they take part in type I and type II immune responses. They also contribute to other non-immunological homeostatic functions like mucus production, tissue regeneration, and angiogenesis. In colorectal cancer (CRC), eosinophils locate in the center of the tumor and in the front of invasion and play an anti-tumoral role. They directly kill tumor cells by releasing cytotoxic compounds and eosinophil extracellular traps or indirectly by activating other immune cells via cytokines. As CRC progresses, the number of infiltrating eosinophils decreases. Although this phenomenon is not fully understood, it is known that some changes in the microenvironmental milieu and microbiome can affect eosinophil infiltration. Importantly, a high number of intratumoral eosinophils is a favorable prognostic factor independent from the tumor stage. Moreover, after immunotherapy, responding patients usually display eosinophilia, so eosinophils could be a good biomarker candidate to monitor treatment outcomes. Finally, even though eosinophils seem to play an interesting anti-tumoral role in CRC, much more research is needed to fully understand their interactions in the CRC microenvironment. This review explores the multifaceted roles of eosinophils in colorectal cancer, highlighting their anti-tumoral effects, prognostic significance, and potential as a biomarker for treatment outcomes.

## 1. Introduction

Traditionally, macrophages and dendritic cells have been seen as the most important myeloid cells in normal and pathologic inflammatory responses in the gastrointestinal tract. However, the paradigm is changing, and other cells like mast cells [1,2], neutrophils [3], and eosinophils [4] are emerging as critical players. This highlights the complexity of interactions between different cell types in the gastrointestinal mucosa.

There are no big differences in the ability of different tissues to recruit neutrophils and mast cells. However, eosinophils have a much higher tropism for the gastrointestinal mucosa than for any other tissue in the body [5,6]. This suggests that eosinophils are important players in the gastrointestinal tract. Unfortunately, they are frequently overlooked in research and clinical practice, leading to limited understanding of their role in physiological and pathological conditions.

Eosinophils fully differentiate in the bone marrow. They have a short life in the blood (approximately 18 h), and they migrate mainly to the gastrointestinal tract and, to a lesser extent, to the respiratory tract [5], where they can live up to 8 days [6].

Eosinophils migrate to the gastrointestinal mucosa through eotaxin 1 (CCL11), which is produced by fibroblasts [7], B cells [8], macrophages, and epithelial cells [9]. Moreover, type-2 innate lymphoid cells (ILC2) also contribute to eosinophil recruitment, releasing IL-5 and IL-13. IL-5 promotes eosinophil differentiation in the bone marrow and makes them more responsive to eotaxin 1. Moreover, IL-13 also induces eotaxin 1 expression in other immune cells, further facilitating eosinophil migration to the gastrointestinal tract [6].

Eosinophils are pivotal cells in the gastrointestinal mucosa. Under normal conditions, they contribute to Peyer patches’ formation, although they are not very abundant when they are fully developed [10,11,12]. This process is essential for efficient antigen sampling and immune surveillance in the gut. Moreover, they also promote mucus production, tissue regeneration, angiogenesis, and the differentiation of T regulatory cells (Tregs) and dendritic cells [10,11,12]. In short, eosinophils play an important role both in immune and non-immune homeostatic functions.

## 2. Role of Eosinophils in Mucosal Immune Responses

Eosinophils can act both as effectors and modulators of mucosal immune responses. As effectors, they are widely known for their role in parasitic infections where they are one of the main cell types involved in the elimination of parasites. The main anti-parasitic mechanisms include the release of granule proteins and antibody-dependent cellular cytotoxicity [13]. Nevertheless, they also contribute to immune responses against bacteria and viruses. Eosinophils detect those microbes through pathogen-associated molecular pattern (PAMP) receptors including TLR1, 2, 3, 4, 5, 6, 7, and 9, as well as through C-type lectin receptors [14,15]. They participate in anti-bacterial responses, releasing cytotoxic proteins and extracellular traps of mtDNA that can directly kill bacteria [16]. Additionally, they can also phagocyte bacteria, although this mechanism is much less relevant than in macrophages or dendritic cells [6]. Eosinophils contribute to antiviral responses by releasing nitric oxide and interferon β (IFN-β) to impair viral replication and IL-8 to recruit neutrophils to the site of infection [17]. Moreover, they release eosinophil cationic proteins (ECPs) and eosinophil-derived neurotoxins (EDNs), which have ribonuclease activity, to degrade RNA viruses and prevent their replication [17]. Noteworthy, eosinophils are able to migrate to lymphoid structures where they upregulate class II MHC and co-stimulatory molecules to present antigens to T lymphocytes and initiate adaptive immune responses. However, they produce less co-stimulation than dendritic cells [18,19].

As modulators of mucosal immunity, they act both in type-1 and type-2 responses [14,20]. In type-1 responses, they secrete type-I IFNs to polarize macrophages to M1 phenotype and promote pro-inflammatory cytokine production, contributing to the elimination of intracellular pathogens [21]. Moreover, they also modulate T follicular helper cells to suppress Th2 responses to further enhance the type-1 immune response [22]. They form lipid bodies (LBs) that serve as a repository of pro-inflammatory cytokines such as tumor necrosis factor α (TNF-α) and lipid mediators like eicosanoids [23]. This allows them to trigger a pro-inflammatory response quickly and efficiently upon encountering a pathogen.

An interesting case is EDN. It recruits, activates, and maturates dendritic cells to promote antigen presentation and activation of T cells [24]. However, depending on the molecular context of the microenvironment, it can contribute to Th1 or Th2 responses by mechanisms that are not completely understood [24].

In type 2 responses, eosinophils polarize macrophages to M2 directly through IL-4 and IL-13 [25] or indirectly by recruiting Th2 lymphocytes to the site of inflammation [6]. Interestingly, they can also induce the switch from M1 to M2 macrophages, inhibiting the IkB/p38 MAPK signaling pathway and promoting the expression of anti-inflammatory molecules such as IL-10 [26]. Under prolonged IFNγ exposure, eosinophils upregulate PD-L1 to inhibit Th1 [27]. Moreover, eosinophils can further contribute to immune tolerance secreting TGF-beta to differentiate CD103+ dendritic cells and Tregs during type-2 responses [28].

Eosinophils also contribute to the homeostasis of the gastrointestinal barrier. Upon activation of TLR2 and TLR4, eosinophils release a mixture of cytokines, including IL-1β and keratinocyte growth factor [11,22,29,30,31]. These changes in the microenvironment promote IgA switching in germinal centers and epithelial integrity [11,22,29,30]. Moreover, eosinophils can also promote T-independent IgA switching, but the exact mechanism is not fully understood [28]. One possibility might be by directly activating B cells through CD40-CD40L interactions and producing BAFF (B-cell activating factor of the tumor necrosis factor family) [11].

In the absence of eosinophils, Peyer patches are smaller and contain fewer cells [12]. Additionally, there is a reduction of IgA+ plasma cells and IgA production, Tregs, CD103+ dendritic cells [28], and ROR-Ɣt ILCs [11]. There was also a decrease in the production of IL-1β, inducible nitric oxide synthase (iNOS), and lymphotoxins α and β [11]. Although eosinophils produce large amounts of A PRoliferation-Inducing Ligand (APRIL) to promote plasma cell survival [31] in its absence, other cell types compensate for APRIL’s production [11]. Overall, this leads to an impaired immune response and increased susceptibility to infection and inflammation in the gastrointestinal tract [11,12,28,31].

Coagulation and fibrinolysis are important in wound healing and tissue remodeling. Likewise, coagulation stages are associated with inflammation and tissue destruction, while fibrinolysis is associated with repair and healing responses [32]. Eosinophils can promote coagulation through tissue factor or thrombin and fibrinolysis through plasminogen activators such as urokinase-type plasminogen activator and tissue-type plasminogen activator [32]. Eosinophils can detect tissue damage with DAMP receptors and contribute to wound healing, releasing transforming growth factor β (TGF-β), TGF-α, fibroblast growth factor (FGF), epidermal growth factor (EGF), platelet-derived growth factor (PDGF), vascular endothelial growth factor (VEGF), and matrix metalloproteinase 9 (MMP9) [15,32].

In a nutshell, depending on their microenvironment, eosinophils in the gastrointestinal mucosa contribute to immune-stimulatory (type-1) or immunosuppressive (type-2) responses. Moreover, they also take part in non-immunological homeostatic functions (mucus production, tissue regeneration, angiogenesis…), which makes them very versatile cells in the microenvironment.

## 3. Recruitment of Eosinophils to the Tumor Microenvironment in Colorectal Cancer

The selective enrichment of eosinophils in gastrointestinal mucosa and its important role in the local microenvironment is a unique feature of the gastrointestinal tract that can affect the origin and progression of colorectal cancer (CRC).

In CRC, eosinophils locate both in the center of the tumor and in the front of invasion, suggesting an active role in the tumor microenvironment. They interact mainly with macrophages in both locations and with neutrophils in the center of the tumor [33]. Noteworthily, the number of tumor-infiltrating eosinophils is a favorable prognostic factor independent from the tumor stage, its histological grading, and vascular invasion [33,34,35,36], although it is usually overlooked in routine clinical practice. Moreover, the number of infiltrating eosinophils decreases as the tumor progresses [37]. After surgical resection, those patients bearing tumors with more that 30 eosinophils/mm developed less metastasis and lived longer [38,39]. Overall, this suggests that their presence has a suppressive effect on tumor growth and invasion, indicating a potential role of eosinophils in CRC prognosis and treatment.

In CRC tumors, infiltrated immune cells and fibroblasts upregulate the production of eotaxin 1 (CCL11), eotaxin 2 (CCL24), eotaxin 3 (CCL26), and ELR^+^ CXC chemokines such as CXCL8 [7]. This drives a significantly higher recruitment and survival of eosinophils in the tumor than in the adjacent normal mucosa [40,41]. Additionally, the necrotic death of tumor cells releases damage-associated molecular patterns (DAMPs) like the high-mobility group box 1 protein (HMGB1) [42] or IL-33 [43]. This HMGB1 binds the receptor for advanced glycation end products (RAGE) in eosinophils and other innate immune cells. In eosinophils it acts as chemoattractant and pro-survival signal and triggers the release of pro-inflammatory cytokines, ROS, and cationic proteins, which can contribute to the anti-tumor response in CRC [42].

## 4. Eosinophils as Effectors in Anti-Tumoral Immune Responses in Colorectal Cancer

**Figure 1:**
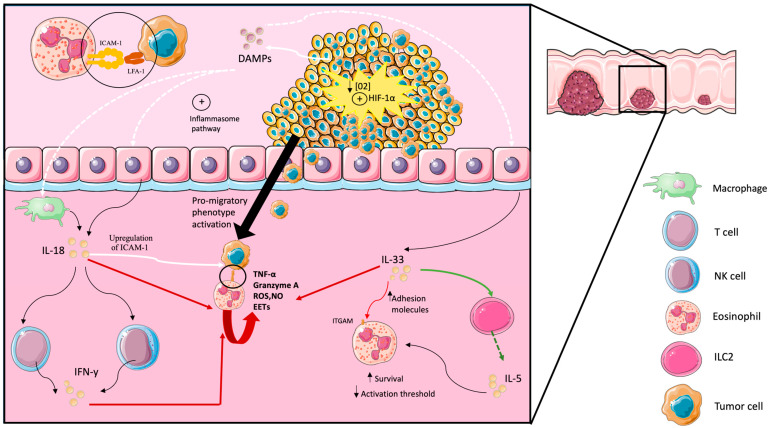
The anti-tumoral role of eosinophils in the colorectal cancer microenvironment.

In early stages of colorectal cancer, tumor cells release damage-associated molecular patterns (DAMPs) and increase the permeability to bacterial products due to a loss in the integrity of the intestinal barrier (Figure 1). Under normal conditions, epithelial cells store IL-33 in intracellular compartments, and in the presence of these bacterial products, they upregulate the expression of IL-33. As the stress sets in, epithelial cells die and release IL-33 to the microenvironment, where it acts as an alarmin to activate immune cells, including eosinophils, in the gastrointestinal mucosa [43]. IL-33 acts on eosinophils to increase the expression of adhesion molecules like integrin alpha M (ITGAM) [44,45] and on ILC2 cells to promote the release of IL-5 and IL-13 [46,47]. Moreover, this IL-5 acts on eosinophils promoting their survival and reducing their activation threshold [48].

Furthermore, in macrophages and epithelial cells, these DAMPs and PAMPs promote the activation of the inflammasome pathway, which releases activated IL-18 [49]. This IL-18 promotes the release of IFN-γ in T and NK lymphocytes leading to further activation of eosinophils [49]. Moreover, this IFN-γ also triggers the upregulation of intracellular adhesion molecule 1 (ICAM-1) in tumor cells, facilitating the adhesion and infiltration of eosinophils into the tumor microenvironment [50]. This expression of ICAM-1 in tumor cells is more prominent in the center of the tumor and in front of invasion [51], which could explain why eosinophils tend to concentrate in those areas. In the center of the tumor, it is potentiated through hypoxic conditions and hypoxia-inducible factor 1α (HIF-1α) activation [51]. Alternatively, in the front of invasion, it interacts with fibrinogen and other components of the extracellular matrix to activate a pro-migratory phenotype [51]. That indicates that the expression of ICAM-1 in tumor cells plays an important role in their adhesion and transmigration.

Eosinophils bind ICAM-1 in the surface of tumor cells through leukocyte function-associated antigen 1 (LFA-1) (CD11a/CD18 integrin) and ITGAM (CD11b/CD18 integrin). When this binding occurs in the presence of IL-18, IL-33, and IFN-γ, eosinophils release TNF-α [52], granzyme A [50], ROS, nitric oxide, and eosinophil extracellular traps (EETs) to kill the tumor cell [42,53]. In the case of the EETs, the mtDNA scaffold is decorated with citotoxic proteins like major basic protein (MBP) and ECP to kill more efficiently [16,54]. Interestingly, in IL-18 KO mice, the frequency and aggressiveness of colorectal tumors is much higher than in control mice [55]. Similarly, the blocking of IL33 signaling impairs the cytotoxic function of eosinophils against colorectal tumor cells [44,45]. However, in melanoma and prostate cancer, IL-18 plays a pro-tumoral role [55], and the same happened with IL-33 in breast cancer [56] and head and neck carcinoma. These differences with IL-18 and IL-33 in other cancers and colorectal cancers may be explained by the difference in the recruitment of eosinophils. Table 1 summarizes the role of eosinophils in antitumoral immune responses in preclinical and clinical models.

## 5. The Cytotoxic Arsenal of Eosinophils

Eosinophils and neutrophils are the main granulocytes recruited to peripheral tissues. However, while neutrophils perform their cytotoxic activity mainly intracellularly (after phagocytosis), eosinophils perform their cytotoxic activity extracellularly by degranulation and the release of EETs [24,56].

Importantly, degranulation and EETs release do not necessarily cause the death of the eosinophil. In the case of EETs, since it is performed with mtDNA, the integrity of the nucleus is not compromised, and the cell remains viable [56]. The main cytotoxic mediators released by eosinophils include MBP, ECP, EPO, EDN, and granzyme A.

MBP and ECP are extremely basic (pI = 11.4 and 10.8 respectively) and toxic. They disrupt the cell membrane, causing chromatin condensation, ROS production, caspase-3 activation, and cell death. Moreover, they alter the functioning of several enzymes, binding to their negatively charged amino acids [24,60]. Importantly, the pores opened by disrupting the cell membrane allow the entrance of other cytotoxic proteins like EDN and granzyme A.

EPO catalyzes the synthesis of hypohalous acids and hypothiocyanous acid. These compounds generate a strong oxidative stress and cause in the target cells lipid oxidation and cell death. Moreover, EPO also catalyzes the nitration of MBP, ECP, and EDN [24].

EDN and granzyme A act intracellularly. EDN is very efficient degrading RNA, becoming highly toxic when arrives to the cytoplasm [52,60,61]. Granzyme A activates caspase-independent cell death and degrades proteins in the nuclear envelope, histones, and other proteins in charge of DNA repair [62].

## 6. Eosinophils as Modulators in Anti-Tumoral Immune Responses in Colorectal Cancer

**Figure 2:**
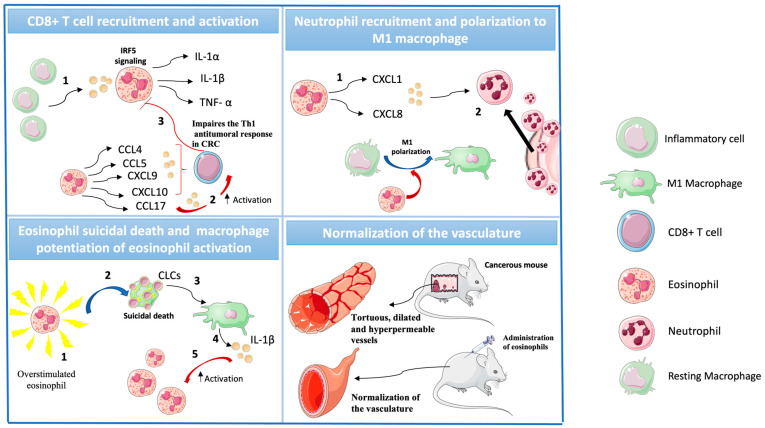
Eosinophils modulate the immune response in the tumor microenvironment. CLC (Charcot Leyden crystals). The numbers indicate the order of the different events.

The role of granulocytes in CRC is more complex than initially expected (Table 2). Under stress or inflammatory conditions, several cell types (including both immune and non-immune cells) release granulocyte-macrophage colony stimulating factor (GM-CSF) to increase the production and activation of myeloid cells. In eosinophils, GM-CSF activates the interferon regulatory factor 5 (IRF5) signaling, which triggers the secretion of several pro-inflammatory cytokines, including IL-1α, IL-1β, and TNF-α. Moreover, after IRF5 activation, eosinophils act on CD8^+^ T cells, promoting their recruitment through CCL4, CCL5, CXCL9, and CXCL10 (Figure 2). Additionally, they activate these CD8^+^ T cells through CCL17, leading to an amplification of the anti-tumoral response. Importantly, blocking of the GM-CSF-IRF5 axis in eosinophils severely impaired the Th1 antitumoral response in CRC, highlighting the important role of eosinophils in antitumoral responses [27,59].

Eosinophils also modulate the myeloid compartment in the tumor microenvironment. They contribute to neutrophil recruitment, releasing CXCL1 and CXCL8 [63]. Once in the tumor microenvironment, eosinophils produce IFN-β [17,21], which contributes to N1 polarization and activation [64]. Noteworthily, eosinophils can also polarize macrophages to M1 even when they are already immunosuppressive tumor-associated macrophages (TAMs) [59]. However, the full mechanism is not completely understood. In short, eosinophils potentiate the antitumoral immune response, modulating both innate and adaptive immune cells.

Even though eosinophils do not necessarily die after activation, if they are overstimulated, they undergo a suicidal death in which they release all their internal content. In this cell death, they release an enigmatic bipyramidal hexagonal crystals known as Charcot Leyden crystals (CLCs) that are composed of eosinophil-derived galectin-10 protein and can be observed in histopathological samples [65]. Although the role of CLCs is far from being completely understood, upon macrophage phagocytosis, they activate the NOD-like receptor family pyrin domain containing 3 (NLRP3) inflammasome and trigger IL-1β release [65]. Noteworthily, the NLRP3 activation also triggers the release of IL-18 [66], which potentiate the activation of other eosinophils.

In CRC, the vasculature plays an important role in the molecular context of the microenvironment. The excessive production of pro-angiogenic factors leads to the formation of abnormal vessels. These vessels are tortuous, dilated, and hyperpermeable. This produces low perfusion, hypoxia, a pH decrease, and high interstitial fluid pressure within the tumor microenvironment. Such impairs the recruitment and survival of anti-tumoral effector cells and promote pro-tumoral immune cells [67]. Noteworthy, in mouse models, the administration of eosinophils promoted the normalization of the vasculature. This increased the amount of CD8^+^ T cells and M1 macrophages and strongly enhanced tumor rejection [59].

**Table 2:** Role of granulocytes in CRC.

Cell Type	Clinical Significance	Modulation	Refs.
Eosinophils	Good prognosis. Circulating eosinopihls are a potential biomarker for improved response to immunotherapy.	Release of cytotoxic proteins, cytokines linked to Th1 responses, ROS. Normalization of the vasculature.	[33,34,35,36,42,50,52,53]
Neutrophils	Poor prognosis	Pro-tumoral: extracellular matrix remodeling, aberrant angiogenesis, and immune suppression.	[68,69,70]
Basophils	Circulating basophils indicate good prognosis	Unclear	[71]
Mast cells	Unclear	Pro-tumoral: extracellular matrix remodeling, aberrant angiogenesis, and immune suppression. Anti-tumoral: release of cytokines linked to Th1 responses, ROS and histamine.	[72,73]

## 7. Microbiome as a Modulator of Eosinophil Responses

The human colon fosters a complex community of microbes (called the microbiome) that is critical for its normal functioning. Under normal circumstances, microbial products contribute to eosinophil recruitment. They can do that directly (acting as chemoattractants) or indirectly (acting on other cells that release eotaxins). In CRC, the barrier dysfunction increases the permeability to microbial products that act like PAMPs.

The activation of TLR2 and TLR5 on eosinophils promotes the upregulation of adhesion molecules, as well as the release of ECP and pro-inflammatory cytokines and chemokines (CXCL1, IL-1β, IL-6, IL-8) [74]. Moreover, LPS activates TLR4 on eosinophils, triggering the release of GM-CSF, TNF-α, and IL-8 [75].

Several studies have associated microbiome imbalance (dysbiosis) with the initiation and progression of CRC [76]. However, it is still unclear if this dysbiosis is a tumor driver or a tumor response [77].

Dysbiosis impacts eosinophils, reducing their recruitment via the downregulation of CD11b, and decreases their granule content and size. Importantly, the normalization of the microbiome restores eosinophil number and phenotype [27,78]. Interestingly, the production of IL-25 in colon epithelium is highly dependent on the microbiome composition [79]. This IL-25 primes eosinophils and works synergistically with other cytokines like IL-33 [80] and increase their anti-tumor activity [81]. Moreover, IL-25 acts on ILC2, promoting the release of IL-5, which contributes to eosinophil recruitment, survival, and activation [82].

## 8. Evasion from Eosinophil Control

**Figure 3:**
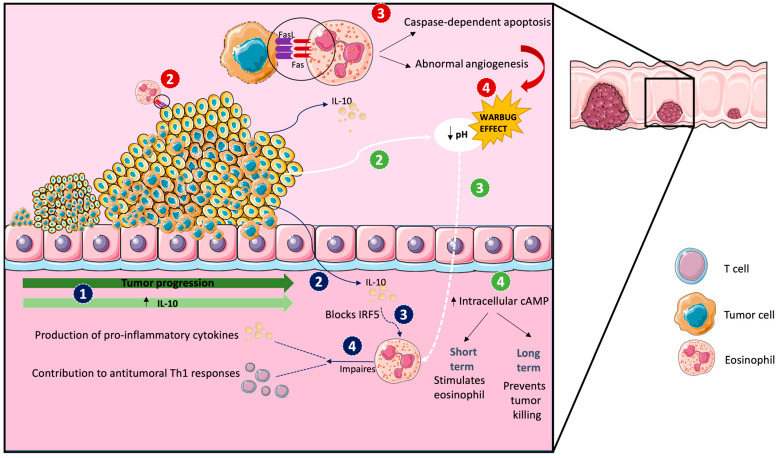
Tumor progression excludes eosinophils from the microenvironment. The numbers indicate the order of the different events. The color of the numbers indicates different independent processes. Number 1 is common to all processes.

The production of eotaxins decreases as the tumor progresses, reducing the recruitment of eosinophils to the tumor microenvironment and potentially impairing the anti-tumor immune response [83]. Moreover, CRC increases the production of IL-10 as it progresses, which is associated with a poorer prognosis [84]. In eosinophils, IL-10 blocks IRF5, which impairs the production of pro-inflammatory cytokines and the contribution of eosinophils to antitumoral Th1 responses [27,78] (Figure 3). This can have pathological consequences as the absence of eosinophils and their impaired activation may lead to a reduced ability to eliminate tumor cells and hinder the effectiveness of cancer treatments.

Another characteristic feature of CRC is the generation of extracellular acidosis due to the Warburg effect, which results in a hostile microenvironment that can inhibit the function of antitumoral immune cells [85]. This increases the intracellular production of cAMP in eosinophils, which stimulates eosinophils in the short term [86]. However, in the long term, the increased cAMP levels can lead to impaired eosinophil migration and reduced anti-tumor immune responses [87]. Moreover, 70% of CRC tumors display a high expression of Fas-L [88], which binds Fas in the eosinophil surface and triggers caspase-dependent apoptosis, resulting in eosinophil depletion within the tumor microenvironment [89]. Noteworthy, eosinophil apoptosis contributes to abnormal angiogenesis [59], which generates more acidosis, forming a feed-forward loop that further impairs eosinophil function and anti-tumor immune responses.

## 9. Potential Clinical Application of Eosinophil Evaluation in Colorectal Cancer

Traditionally, the tumor-node-metastasis (TNM) system has been the gold standard for tumor grading and prognosis in CRC patients. This system evaluates the size of the primary tumor, the presence of tumor cells in lymph nodes, and the presence of distal metastasis. However, recently, a new method based on immune infiltration (immunoscore) was developed. The immunoscore analyzes the abundance of cytotoxic T lymphocytes in the center and the periphery of the tumor and provides a more comprehensive assessment of the immune response within the tumor microenvironment [90]. Interestingly, the immunoscore has a superior prognostic value and is independent from TNM [91]. Moreover, it can predict the response to immunotherapy [91]. Therefore, currently, immunoscore and TNM are used together in clinical practice [91].

One of the limitations of immunoscore is that it only analyzes T cells. As discussed before, eosinophils play an important antitumoral role and are an independent prognostic factor from the TNM system and other clinical factors [33,34,35,36,37,38,39]. Therefore, we believe that the inclusion of eosinophils in the immunoscore could help to improve patient prognosis.

Additionally, eosinophils can be used to monitor treatment response. After immunotherapy with IL-2 and IL-4, the patients that responded to treatment developed eosinophilia, indicating a successful immune response against the tumor [92,93,94]. Interestingly, those circulating eosinophils had higher anti-tumoral activity than normal eosinophils [93]. This eosinophilia also happens after immune checkpoint therapy and correlates with better control of the disease [95]. However, these studies included small cohorts of patients, and further research is needed to validate the use of eosinophils as a biomarker for treatment response in larger patient populations.

Noteworthily, this beneficial effect of eosinophils in treatment response is not limited to CRC and was observed also in melanoma [96], breast cancer [97,98], soft tissue cancer [35], and gastric cancer [35].

## 10. Future Directions

Unfortunately, there is still a lot to learn about eosinophils in CRC and their specific role in the gastrointestinal tract, particularly about their interaction with other immune cells, tumor cells, and microbiome. So far, one of the biggest challenges of working with eosinophils has been that they are very sensitive to shear stress. Moreover, the breakdown of their granular contents during isolation and culture destroys their cellular content, making it difficult to study their functional properties accurately. Therefore, the development of new techniques and methods to study eosinophils in their native context is crucial for a better understanding of their role in gastrointestinal health and disease.

Eosinophils produce IFN-γ in the gastrointestinal mucosa [81], and that contributes to N1 and M1 polarization of neutrophils and macrophages respectively. Moreover, in murine models lacking lymphocytes, eosinophils become the main producers of IFN-γ [99]. Therefore, it would be interesting to see how much of the IFN-γ in the CRC microenvironment is produced by eosinophils.

The evasion of eosinophil control in CRC is still poorly understood. In other cell types like macrophages or neutrophils, the tumor polarizes them to wound-healing phenotypes that contribute to tumor progression [68,88]. Although eosinophils can polarize toward a wound-healing phenotype, they are excluded from the tumor microenvironment. Why do CRC tumors exclude the eosinophils instead of polarizing them to a wound-healing phenotype?

The microbiome can modulate the recruitment and phenotype of eosinophils [20,27]. However, we do not know which species control this process and which are the most relevant mediators. As CRC tumors progress, dysbiosis increases [76], and the number of eosinophils decreases [37]. Therefore, understanding the interactions between the microbiome and the eosinophils will show if dysbiosis is a critical mechanism for avoiding eosinophil control.

For clinical practice, we need more studies to see if eosinophils are useful as biomarkers to monitor treatment outcomes. Moreover, it would be interesting to see if the inclusion of eosinophils in the immunoscore improves its prognostic value.

## 11. Conclusions

Eosinophils have an important antitumoral role in CRC. They can directly kill tumor cells or modulate other immune cells to promote an antitumoral response. Their presence in the tumor microenvironment is an indicator of a good prognosis independent of the TNM stage. Moreover, eosinophilia with an increased antitumoral phenotype is common after immunotherapy. Despite their important antitumoral role in CRC, there is a lot to discover about their relationship with other immune cells and the microbiome. Finally, we also need more studies to understand how CRC evades CRC control.

## Figures and Tables

**Table 1:** Summary of preclinical and clinical trials and critical parameters for evaluating Eosinophils as effectors in anti-tumoral immune responses in colorectal cancer.

Setup of CRC Treatment	Model	Treatment Parameters	Results	Ref.
C57BL/6 mice, IL-5–transgenic, eosinophil-deficient mice (PHIL), BALB/c. Eo-Cre × Irf5 ^fl/f^, mice, and Eo- Cre × Csf2rb^fl/fl^ were injected with MC38 CRC cells derived from B57BL/6 mice or with the CT26 cell line derived from BALB/c mice. Apc^Min/+^ mice were treated with anti-IL5.	- Syngenic ectopic murine models of colorectal cancer - 240 CRC patients	Tumor weight, volume, leukocyte infiltration, and RNA expression profile	Eosinophil activation and migration to the tumor site required GM-CSF signaling, with GM-CSF-activated eosinophils driving CD4^+^ and CD8^+^ T cells activation and infiltration, which inversely correlated with the tumor state.	[57]
C57BL/6 mice and CD3-IL5 transgenic mice and Apc^Min/+^ mice were injected intraperitoneally with azoxymethane (AOM) and dextran sodium sulfate (DSS) or were injected with MC38.	Murine model of inflammation-induced colorectal cancer and orthopic model	Status and number of tumors versus percentage of eosinophilic infiltrate, size and number of tumors, quantitative assessment of tumor load in adenoma and transcriptome, and proteomic analysis of intratumoral eosinophil	Intratumoral eosinophils had a phenotype, which was associated with IFN-γ signaling. IFN-γ potentiated the eosinophil-mediated killing of colorectal cancer (CRC) cells by the release of reactive oxygen species, mitochondrial DNA, and nitric oxide.	[58]
Stage I and II patients did not receive adjuvant therapy, whereas stage III patients were given 5-fluorouracil/folinic acid-based chemotherapy.	381 colorectal cancer patients	TNM classification, tumor cell differentiation, vascular invasion and tumor budding	Increasing peritumoral and intratumoral eosinophil counts were associated with favorable tumor parameters (lower T and N classification), progression-free and cancer-specific survival, and although the peritumoral eosinophil count correlated with the intensity of the overall inflammatory cell reaction, it was independently associated with the outcome.	[34]
For the heterotopic CRC model, CT26 cells were injected subcutaneously into the flank of BALB/c or ΔdblGATA-1 mice. When tumors were palpable, mice were treated with IL-33. For colitis-associated CRC, model mice were injected wirh AOM and DSS.	Heterotopic CRC tumor engraftment model and colitis-associated CRC model.	Tumor area, volume, weight, leukoyite infiltration, eosinophil infiltration, and cell viability	Reduction in tumor growth was significantly enhanced when eosinophils were activated by IL-33, and the degranulation of eosinophils seemed to be the mechanism that contributed to the IL-33 dependent anti-tumoral effects.	[45]
C57BL/6N mice and transgenic Foxp3.LuciDTR-4 BAC mice (DTR4) were injected with the MC38 aenocarcinoma cell line. For the depletion of T_reg_ cells, transgenic Foxp3.LuciDTR-4 BAC mice received intraperitoneal injection of diphtheria toxin. For the depletion of eosinophils, anti Siglec-F was injected.	Heterotopic CRC tumor engraftment model	Tumor size, % survival, epsinophil infiltration, vessel normalitation, and % of migration	Eosinophils migrated preferently into tumors and les into other tissues such as the lymphoid organs or liver. Activated tumor-infiltrating eosinophils produced large amounts of chemokines, such as CCL5, CXCL9, and CXCL10, that recruited co-transferred CD8^+^ T cells to the tumor, which resulted in tumor rejection and prolonged survival. Eosinophil infiltration also normalized tumor vasculature and macrophage polarization.	[59]
